# Reproductive robustness differs between generalist and specialist maternal rabbit lines: the role of acquisition and allocation of resources

**DOI:** 10.1186/s12711-014-0073-5

**Published:** 2015-01-17

**Authors:** Davi Savietto, Nicolas C Friggens, Juan José Pascual

**Affiliations:** Instito de Ciencia y Tecnología Animal (ICTA), Universitat Politècnica de València, Camino de Vera s/n, 46022 Valencia, Spain; INRA, UMR 791 Modélisation Systémique Appliquée aux Ruminants (MoSAR), 16 rue Claude Bernard, F-75231 Paris, France; AgroParisTech, UMR 791 Modélisation Systémique Appliquée aux Ruminants (MoSAR), 16 rue Claude Bernard, F-75231 Paris, France

## Abstract

**Background:**

Farm animals are normally selected under highly controlled, non-limiting conditions to favour the expression of their genetic potential. Selection strategies can also focus on a single trait to favour the most ‘specialized’ animals. Theoretically, if the environment provides enough resources, the selection strategy should not lead to changes in the interactions between life functions such as reproduction and survival. However, highly ‘specialized’ farm animals can be required for breeding under conditions that differ largely from selection conditions. The consequence is a degraded ability of ‘specialized’ animals to sustain reproduction, production and health, which leads to a reduced lifespan. This study was designed to address this issue using maternal rabbit lines. A highly specialized line with respect to numerical productivity at weaning (called V) and a generalist line that originated from females with a long reproductive life (called LP) were used to study the strategies that these lines develop to acquire and use the available resources when housed in different environments. In addition, two generations of line V, generations 16 and 36, were available simultaneously, which contributed to better understand how selection criteria applied in a specific environment changed the interplay between functions related to reproduction and survival.

**Results:**

We show that, under constrained conditions, line LP has a greater capacity for resource acquisition than line V, which prevents excessive mobilization of body reserves. However, 20 generations of selection for litter size at weaning did not lead to an increased capacity of nutrient (or resource) acquisition. For the two generations of line V, the partitioning of resources between milk production, body reserves preservation or repletion or foetal growth differed.

**Conclusions:**

Combining foundational and selection criteria with a specific selection environment resulted in female rabbits that had a different capacity to deal with environmental constraints. An increased robustness was considered as an emergent property of combining a multiple trait foundational criterion with a wide range of environmental conditions. Since such a strategy was successful to increase the robustness of female rabbits without impairing their productivity, there is no reason that it should not be applied in other livestock species.

## Background

In farmed livestock, robustness (as defined by Knap [1]) represents the ability of an animal’s genotype to maintain a good production level while maintaining all other life functions in a wide variety of environmental conditions (i.e. food quality, temperature, pathogen load, management, etc.). Based on this definition, robust animals, with respect to various life functions, may be considered as ‘generalist animals’. However, intensive selection of farm animals to increase productive traits has resulted in specialized breeds and strains as for example broilers [2,3] and dairy cows [4] and also for pigs [5], hens [6] and rabbits [7,8].

North American Holstein-Friesian dairy cows, an example of a highly specialized cattle breed for milk yield, prioritize milk production [9] at the expense of fertility [10]. Other examples of undesired effects in response to selection have been described for different species i.e. pigs [11,12], poultry [13,14] and rabbits [15,16], which has led to the general perception that selection degrades the ability of animals to simultaneously sustain production, reproduction and health [17,18]. Nevertheless, artificial selection of high producing animals does not necessarily entail the emergence of negative effects as shown for dairy cows [19] or rabbits [20,21], and breeds and strains that can sustain production, fertility and health under different environments (i.e. ‘generalist’) can be obtained. This is of special interest because it indicates that it is possible to select animals that are able to balance production, reproduction and health. However, the amount of information on the consequences of selecting ‘generalist’ farm animals with respect to their performance across environments, especially constrained environments, is currently insufficient.

Theoretically, if animals are selected under non-limiting conditions, responses to selection can be achieved without modifying the interplay between life functions, whereas under limiting conditions, this interplay is affected [22,23]. However, this theory is not useful to provide insights into the consequences of selecting highly specialized animals in relatively good environments on their ability to cope with poor environments. Our study was designed to address this issue, using two maternal rabbit lines: a highly specialized line (i.e. line V) in terms of numerical productivity (i.e. litter size at weaning), and a more generalist line (i.e. line LP) that was founded for reproductive longevity and then selected for litter size at weaning. In addition, for line V, two generations 16 and 36, were available simultaneously. Lines V and LP have been shown to differ in their ability to maintain litter size in the presence of constraints [20,24], and also in the strategies used to attain the breeding objectives (e.g. use of body reserves [25] and shape of lactation curve [26]).

To evaluate the capacity of resource acquisition of these lines with different selection backgrounds and their resource allocation strategies, three environmental conditions were set up. Our aim was to study the ability of these maternal rabbit lines to acquire (feed intake) and allocate (litter size, milk production and body condition) the resources available in markedly different environments.

## Methods

### Rabbit lines and selection history

#### Specialist maternal rabbit line (line V)

The specialist maternal rabbit line named line V was established at the Universitat Politècnica de València in 1981 by crossing the progeny of four specialized maternal rabbit lines. After three generations of random mating by avoiding mating between close relatives, selection to increase the number of kits weaned per litter started [27]. Over generations, the effective population size was maintained at 120 females and 25 males. A large number of males were used to keep a low level of inbreeding. For each generation, at least one male offspring per sire was retained and the mating between relatives that shared a grand-parent was avoided. Selection was also conducted in non-overlapping generations of nine months. For each generation, young females were weaned at 28 days, and those that reached the age of 4.5 months were mated. After parturition, mating was attempted on day 11 to reach a reproductive cycle of 42 days, and females were culled only after three consecutive failures due to infertility. To preserve the genetic material, the Universitat Politècnica de València rabbit selection programme has a cryopreserved control population. Every two or three generations of selection, embryos from a representative sample of the best matings (for each male, two or more straws of embryos are cryopreserved) are recovered and vitrified. Recently, this line V reached generation 36.

Since selection began, no substantial improvement in the selection environment of line V was made (Baselga, personal communication). Animals are housed in flat deck indoor cages, with free access to water and commercial pelleted diets (minimum of 15 g of crude protein per kg of dry matter (DM), 15 g of crude fibre per kg of DM, and 10.2 MJ of digestible energy (DE) per kg of DM). The photoperiod is set to provide 16 h of light and 8 h of dark, and the room temperature is regulated to keep temperatures between 10°C and 28°C. Rooms are cleaned and disinfected every week and the animals are vaccinated against rabbit haemorrhagic diseases and myxomatosis. Sick animals are also culled (e.g. due to respiratory disorders, pasteurellosis, sore hocks, etc.). No animals are culled for low productivity.

To evaluate the specialization process in response to the long-term selection design, on the criterion of reproduction only, both generations 16 and 36 of line V were used (hereafter referred to as V16 and V36). The parents of the V16 females used in this study, stored as vitrified embryos, were thawed and transferred to females from another line, also selected for litter size at weaning (line A [27,28]). After one generation without selection, to avoid the environmental maternal effect, 72 young V16 females were obtained and compared with 79 V36 females. For detailed information on the cryopreservation and embryo transfer techniques used in this study see Vicente et al. [29] and Besenfelder and Brem [30], respectively.

#### Generalist maternal rabbit line (line LP)

The generalist maternal rabbit line named line LP was established between 2002 and 2003 by applying a very high selection intensity (i.e. two to five females in one thousand were selected) to obtain females with a long reproductive lifespan (i.e. at least 25 parturitions averaging a minimum of 7.5 kits born alive per parturition). To identify productive females with a long life expectancy, three screening steps were performed in commercial rabbit farms that were located over the whole of the Iberian Peninsula. In the first screening, 15 females were identified and transferred to the facilities of the Universitat Politècnica de València. They were inseminated with semen from males of generation 27 from line V (the current generation in 2002). Fifteen ½ LP and ½ V males were obtained from 12 females. These males were used to inseminate a new set of 15 females selected from a second screening, generating a total of 17 ¾ LP and ¼ V males. These males were then used to inseminate a final group of 32 females from a final screening. A total of 32 males and 42 females ($$ {\scriptscriptstyle \raisebox{1ex}{$7$}\!\left/ \!\raisebox{-1ex}{$8$}\right.} $$ LP and $$ {\scriptscriptstyle \raisebox{1ex}{$1$}\!\left/ \!\raisebox{-1ex}{$8$}\right.} $$ V) were produced from 30 females that constituted the generation 0 of line LP. From that time on, line LP was selected to increase litter size at weaning (currently this line has reached generation 6) under similar conditions as those applied for line V. The direct consequences of the multi-trait criteria used to select the founders of line LP, regardless of the environmental conditions, are that the resulting animals have a long productive lifespan (35 days more than line V [21]), a constant reproductive effort through life (the maximum reproductive performance of line V is reached at parity four [20]) and a better innate immune response in constrained conditions [31,32].

### Environmental conditions

To evaluate the resource acquisition capacity and resource allocation strategy that were derived from the foundational criteria and selection histories of the three genetic types, three environmental conditions were set up by applying various room temperatures and/or diet compositions: (1) a control environment (NC) that combined normal (N) room temperatures (daily variation from 18°C to 24°C) and a control (C) diet that was formulated to achieve 11.6 MJ of DE per kg of DM, 126 g of digestible protein per kg of DM and 169 g of acid detergent fibre per kg of DM; (2) a heat environment (HC) that combined high (H) room temperatures (using a climatic chamber that was designed to produce a daily sinusoidal temperature curve from 25°C to 35°C; detailed specifications are in [33]) and the above diet C; and (3) a nutritionally constrained environment (NF) that combined normal room temperatures (N) with a low-energy fibrous (F) diet that was formulated to achieve 9.1 MJ of DE per kg of DM, 104 g of digestible protein per kg of DM, and 266 g of acid detergent fibre per kg of DM. The detailed composition of the diets is available in [34].

Housing facilities (cages, feeders, drinkers, nest box, etc. and their display), photoperiods (16 h of light and 8 h of dark) and reproductive management were identical for all environments.

### Experimental procedures

At first parturition, 236 LP, V16 and V36 females were randomly allocated to one of the three environments (NC, HC, or NF) in a 3 × 3 factorial design i.e. LPNC = 26, LPHC = 31, LPNF = 28, V16NC = 22, V16HC = 31, V16NF = 19, V36NC = 25, V36HC = 29, and V36NF = 25). The number of animals initially housed under each environment depended on the availability of animals in the selection nucleus. Of these 236 females, 191 reached third parturition i.e. LPNC = 21, LPHC = 26, LPNF = 24, V16NC = 17, V16HC = 23, V16NF = 16, V36NC = 19, V36HC = 21, and V36NF = 24.

Females were subjected to a semi-intensive reproductive rhythm of 42 days and monitored until the third parturition. They were inseminated on day 11 post-parturition and their litters were weaned on day 28 (these are normal procedures within a selection nucleus), which controlled confounding of the resource acquisition of dams. Females that had not conceived on day 11 were re-inseminated 21 days later, and this was repeated for a maximum of three attempts; then non-pregnant animals were culled for low fertility.

Litter size (number of kits born in total and live born kits) and litter weight (weight in g of the kits born in total and live born kits) were monitored at birth. Litters were then standardized at nine kits in the first lactation and 10 kits in the second lactation, so that the three genetic types shared a similar lactation burden. Subsequently, dead kits were not replaced, and both litter size and weight were monitored at weaning. Milk yield (g/d) was measured four days a week during the first three weeks of lactation by weighing females before and after nursing. During the whole experiment, females were fed *ad libitum.* Dry matter intake was measured weekly during the first three weeks of lactation, and during the weaning to parturition interval, which varied according to the females’ real reproductive rhythm. The females’ digestible energy intake (MJ/d) was calculated based on DM intake and apparent digestible coefficients of gross energy that were obtained in a digestibility trial for LP, V16, and V36 females under the NC, HC, or NF environments (values available in [34]).

Female body condition was assessed by measuring live weight (at 0, 7, 14, 21 and 28 days post-parturition) and perirenal fat thickness (PFT) (at 0, 14 and 28 days post-parturition). PFT (mm) was measured by ultrasonography according to Pascual et al. [35].

### Data management and statistical analyses

The data used in this work has already been partially used elsewhere [25,26,34]. To avoid any influence from non-controlled factors that may affect resource acquisition capacity and/or resource allocation of the three genetic types, data for 45 of the 236 housed females that did not reach third parturition were discarded. Reasons for culling 17 females were low fertility (LPHC = 2, LPNF = 1, V16NC = 1, V16NF = 1 and V36NC = 1), retained foetus (LPNC = 1 and V36NC = 1) or diseases (i.e. pasteurellosis: LPNF = 2, V16HC = 2, V16NC = 2 and V16NF = 2 and colibacillosis: V16HC = 1). Another 28 females were found dead (LPHC = 3, LPNC = 3, LPNF = 2, V16HC = 5, V16NC = 2, V36HC = 8, V36NC = 4, and V36NF = 1).

Prolificacy of LP, V16 and V36 females was assessed, under each environment, as the cumulative number of kits produced during the second and third parturitions. Since litters were standardized at birth, the cumulative number and the average weight of weaned kits represented, to some extent, the female’s maternal ability. The resource acquisition capacity was measured as the total DM or DE intake during the first three weeks of lactation and during the period between weaning and parturition (regardless of total intake and number of days between weaning and parturition; i.e. we considered the real weaning to parturition interval of each female) during the first two reproductive cycles. Female live weight and PFT are the average values measured during the first two reproductive cycles (i.e. between first and second parturitions and between second and third parturitions).

Statistical analyses were performed using the general linear model function of R software [36] and the least square means were computed using the lsmeans package [37]. The model used to analyse the cumulative numbers of kits born in total, live born kits, and weaned kits, as well as the individual weight of kit weaned included an effect for environment (E: HC, NC and NF), genetic type (G: LP, V16 and V36) and the interaction between both:

1 $$ {\mathrm{Y}}_{\mathrm{i}\mathrm{j}} = {\mathrm{E}}_{\mathrm{i}}+{\mathrm{G}}_{\mathrm{j}}+\left({\mathrm{E}}_{\mathrm{i}} \times {\mathrm{G}}_{\mathrm{j}}\right) + {\mathrm{e}}_{\mathrm{i}\mathrm{j}}. $$

The model used to analyse the weight of individual kits (for kits born in total and live born kits) also included the total number of kits born (KT) as a covariate:

2 $$ {\mathrm{Y}}_{\mathrm{i}\mathrm{jk}} = {\mathrm{E}}_{\mathrm{i}} + {\mathrm{G}}_{\mathrm{j}}+\left({\mathrm{E}}_{\mathrm{i}} \times {\mathrm{G}}_{\mathrm{j}}\right) + \mathrm{K}{\mathrm{T}}_{\mathrm{k}}+{\mathrm{e}}_{\mathrm{i}\mathrm{jk}}. $$

The model used to analyse intakes of DM and DE, live weights and PFT included parturition order (PO: first or second) as a fixed effect:

3 $$ {\mathrm{Y}}_{\mathrm{i}\mathrm{jk}} = {\mathrm{E}}_{\mathrm{i}} + {\mathrm{G}}_{\mathrm{j}}+\left({\mathrm{E}}_{\mathrm{i}} \times {\mathrm{G}}_{\mathrm{j}}\right) + \mathrm{P}{\mathrm{O}}_{\mathrm{k}} + {\mathrm{e}}_{\mathrm{i}\mathrm{jk}}. $$

Finally, the model used to analyse milk yield incorporated the average number of kits during lactation (KL) as a covariate:

4 $$ {\mathrm{Y}}_{\mathrm{i}\mathrm{jk}} = {\mathrm{E}}_{\mathrm{i}} + {\mathrm{G}}_{\mathrm{j}}+\left({\mathrm{E}}_{\mathrm{i}} \times {\mathrm{G}}_{\mathrm{j}}\right) + \mathrm{K}{\mathrm{L}}_{\mathrm{k}} + {\mathrm{e}}_{\mathrm{i}\mathrm{jk}}. $$

**Figure 1:**
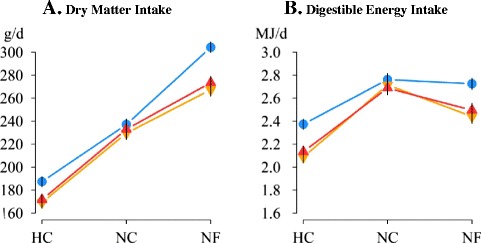
**Resource acquisition capacity of LP, V16 and V36 females housed under different challenging conditions.** Average daily dry matter (g/d) and digestible energy (MJ/d) intakes from first to third parturition (except from day 21 to day 28 of lactation) are in panels **A** and **B**, respectively. Blue: LP, orange: V16 and red: V36; heat (HC), normal (NC) and nutritional (NF) challenging conditions; vertical lines represent the standard error of means.

**Figure 2:**
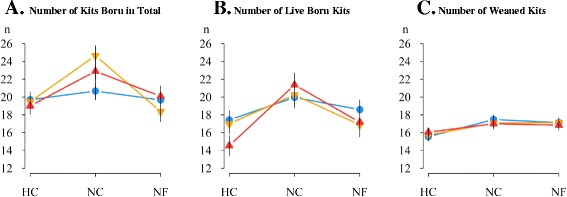
**Litter sizes of LP, V16 and V36 females housed under different challenging conditions.** Cumulative number of kits born in total and number of live born kits in the second and third parturition are in panels **A** and **B**, respectively. Cumulative number of kits weaned during the first two lactations is also shown **C**. At birth, litters were standardized at 9 kits in the first lactation and at 10 kits in the second lactation (true value: 18.9 ± 0.7 kits per female; mean ± s.d.) with dead kits not replaced. Blue: LP, orange: V16 and red: V36; heat (HC), normal (NC) and nutritional (NF) challenging conditions; vertical lines represent the standard error of means.

**Figure 3:**
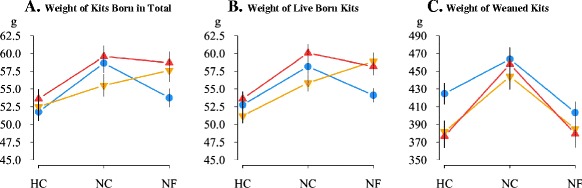
**Weight of litters of LP, V16 and V36 females housed under different challenging conditions.** Average individual weights (g) of kits born in total, live born kits and weaned kits are in panels **A**, **B**, and **C**, respectively. Blue: LP, orange: V16, and red: V36; heat (HC), normal (NC) and nutritional (NF) challenging conditions; vertical lines represent the standard error of means.

**Figure 4:**
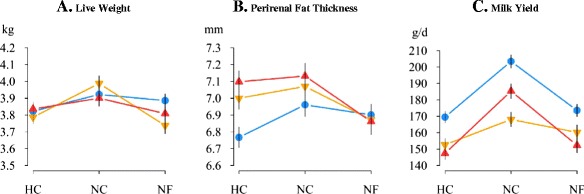
**Body condition and milk yield of LP, V16 and V36 females housed under different challenging conditions.** Average live weight (kg), perirenal fat thickness (mm), and daily milk yield (g/d in the first three weeks) of the first two reproductive cycles are in panels **A**, **B**, and **C**, respectively. Blue: LP, orange: V16 and red: V36; heat (HC), normal (NC) and nutritional (NF) challenging conditions; vertical lines represent the standard error of means.

**Figure 5:**
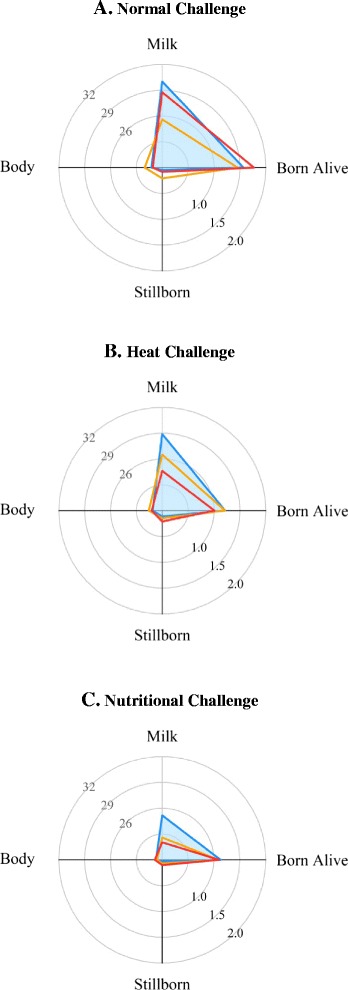
**Radial plots showing the percentage of total energy acquired and assigned to milk production, litter and body.** Data on milk (MJ), litter (MJ of stillborn and live born kits), and body energy (MJ) of LP (blue area), V16 (orange lines), and V36 (red lines) females housed under normal, heat or nutritional challenging conditions are in panels **A**, **B**, and **C**, respectively. Energy content of milk was calculated assuming 8.5 MJ per kg of milk [38]. Energy content per kg of kit was assumed to be 3.4 MJ [39] and energy content in the body (MJ) was calculated from equation (5) developed by Pascual et al. [35]. The scale used for milk energy is different from the other axis (ranging from 20 to 32%). The average standard error of the means for each component trait was: 0.05 for Body, 1.16 for Milk, 0.10 for Live Born and 0.04 for Stillborn. The standard error of means for the different lines and environments are in Figures 1, 2, 3 and 4.

Reaction norms were used to evaluate the overall environmental sensitivity according to foundational criteria or selection histories (Figures 1, 2, 3 and 4). Data on the percentage of energy acquired that was allocated to milk synthesis, litter production (stillborn kits and live born kits) and body growth are presented in a radial plot (Figure 5) to compare the different allocation strategies used by each genetic type under the different environmental constraints. For each female, energy acquired (MJ) was calculated as the sum of energy intake during the first three weeks of lactations one and two (excluding data of the fourth week) plus the energy intake recorded during the weaning to parturition intervals. The amount of energy in milk was calculated as the total amount of milk produced in the first three weeks of lactations one and two assuming that the energy content of milk was 8.5 MJ per kg of milk [38]. Body energy was calculated as the cumulative loss of body energy during lactations one and two based on carcass energy content at parturition and at weaning that was estimated with the equation developed by Pascual et al. [35]:

5 $$ \mathrm{Body} = 2.51 + 0.012 \times \mathrm{L}\mathrm{W} + 0.00018 \times {\mathrm{PFT}}^3, $$

where Body is the estimated body energy (MJ), LW is the live weight (g) and PFT is the perirenal fat thickness (mm). Finally, energy content of the litter was calculated as the total weight of stillborn and live born kits in the second and third parturitions assuming 3.4 MJ per kg of kits produced [39]. Plotted values are lsmeans of a statistical model equal to model (1).

## Results

### Resource acquisition capacity

Average daily DM and DE intakes of LP, V16 and V36 females under normal (NC), heat (HC), and nutritional (NF) challenging environments are in Figures 1A and 1B, respectively. As expected, NF was associated with an increased DM intake but a limited DE intake, whereas HC depressed both DM and DE intakes. Performance results revealed that LP, V16, and V36 females responded differently to the constrained conditions. Under the HC and NF environments, LP females displayed a greater resource acquisition capacity (as shown by energy intake; P <0.05) than both V16 and V36 females. Under normal conditions, DM intake was not significantly different between LP, V16 and V36 females. However, LP females were able to ingest on average 0.27 MJ of DE/d more than V females (P <0.05) under the HC environment. Under the NF environment, LP females had a higher DM intake than V16 and V36 females (on average +33.9 g DM/d; P <0.05), which resulted in LP females having a similar DE intake to that observed in normal conditions. The decrease in DE intake due to exposure to the nutritional challenge was higher for V16 (−0.28 MJ DE/d compared to V16 under the NC environment; P <0.05) than for V36 females (−0.19 MJ DE/d compared to V36 females under the NC environment; P = 0.17).

### Resource allocation strategies

At first parturition, LP, V16 and V36 females had on average a total number of kits born equal to 9.3, 9.5 and 10.3, respectively, with 8.6, 8.4 and 9.3 live born kits, respectively. There were no statistically significant differences between the lines (the pooled standard error of means was 0.57 for total number of kits born and 0.68 for number of live born kits).

Constrained conditions did not affect the reproductive performance of LP females (Figures 2A and 2B), which had similar litter sizes at birth under all environments (e.g. 10.4, 9.9 and 9.9 total kits born per parturition under the NC, HC and NF environments). However, the constrained environmental conditions affected the reproductive performance of V females and this effect differed between generations 16 and 36. Under the HC and NF environments, V16 females had a much lower total number of kits born (−2.6 and −3.2 kits per parturition under HC and NF, respectively; P <0.01) compared to the NC environment but the decrease in number of live born kits was less pronounced (−1.6 and −1.7 kits per parturition under HC and NF, respectively; P = 0.67). In contrast, under the HC and NF environments, V36 females were more able to maintain the total number of kits born (−2.0 and −1.4 kits per parturition under HC and NF, respectively; P *= *0.35) than the number of live born kits (−3.4 kits per parturition; P = 0.003 and −2.1 kits per parturition; P = 0.24 under HC and NF, respectively). After litter standardization at birth, no difference in the number of kits weaned was observed between genetic type or environmental constraint (on average 9.5 kits per parturition; Figure 2C).

Reaction norm plots for average individual weights of kits at birth and weaning are in Figure 3. Productive females with a long life expectancy gave birth to kits with a lower weight under constrained conditions than under normal conditions (on average −6.9 and −4.7 g for total kits born and live born kit, respectively; P <0.05). The individual weights at birth of V16 kits under constrained and normal conditions were not significantly different. However, although the individual weights of V36 kits at birth were similar under NF and NC environments, they were significantly smaller under the HC environment (−6.0 and −6.5 g for total kits born and live born kits, respectively; P <0.05). Across environments, the individual weight of kits weaned by LP females was on average 26.7 g greater than that of kits weaned by V16 and V36 females (Figure 3C).

Average live weight, PFT and daily milk yield of females are in Figure 4. Only V16 females displayed a significant decrease in live weight under constrained conditions (−200 and −250 g under HC and NF compared to NC, respectively; P <0.05). Response in PFT to the different environments varied between genetic types. Although PFT was similar for all genetic types under NC and NF environments, LP females showed a greater decrease in PFT under the HC environment than under the NC environment (−0.28 mm; P <0.05). Regardless of environmental constraints, LP females produced more milk than V16 and V36 females (on average +21.1 g/d). Constrained conditions resulted in lower milk yields compared to normal conditions for both LP (−34.0 and −29.9 g/d under HC and NF, respectively; P <0.001) and V36 females (−38.0 and −33.1 g/d under HC and NF, respectively; P <0.001). In contrast, V16 females had similar milk yields under all environmental conditions.

## Discussion

### Selection context and origin of maternal rabbit lines

The context in which the founder LP females originated and the selection criteria and environmental conditions applied to select line V are important to understand the distinct resource acquisition capacities and allocation strategies used by these lines to attain their fitness. It is also important to note that generally, under commercial conditions, healthiness and prolificacy (i.e. fertility, litter size and maternal ability) are the main factors that condition female fitness and lifespan.

The LP line was established by selecting females from different commercial farms in the Iberian Peninsula that had at least 25 parturitions with a minimum number of 7.5 live born kits per parturition [21], which led to very robust females [20,24,25,31,32]. This robustness is the consequence of the seasonal and punctual fluctuations in the environmental conditions that occur in commercial farms. In order to avoid culling and ensure a long productive lifespan, females must have a good long-term adaptive capacity to adequately face these environmental constraints across many parturitions. Here, we also observed that LP females displayed greater reproductive stability under constrained environments (i.e. litter size was maintained) than V16 and V36 females, which is why they are perceived as generalist animals.

Line V was founded 30 years ago from four specialized maternal rabbit lines [27] and, ever since, has been selected to increase litter size at weaning (it has now reached generation 36) in a context where the intergenerational change (9 months) limited the females’ productive life and the time span during which they demonstrate their genetic potential. In addition, the interval between parturitions of 42 days that was used (females are mated on day 11 post-parturition), with litters weaned on day 28 post-parturition, places selection pressure on females to adequately nurse the current litter while the future litter is developing in utero. In this sense, V females have been selected to cope with the short-term nutritional stress involved in weaning the greatest number of kits possible. Therefore, during the selection process, V females must have adopted specific strategies to be selected. Our study shows that the resource usage patterns to sustain litter size at weaning differ between the two generations of line V i.e. V16 and V36, which indicates that a specialization process is ongoing.

### Resource acquisition capacity

The constrained conditions of HC and NF environments were designed to limit the energy intake of rabbit females. Hot temperatures impair energy intake; i.e. even when fed with a high-energy-density diet, the animals are unable to meet their daily requirements [40]. High-fibrous low-energy diet act differently; females increase their feed intake in an attempt to satisfy their daily energy requirements but are physically limited [41]. The designed constraints worked as expected.

As hypothesized, the resource acquisition capacity of rabbit females differed depending on foundational criteria and selection histories of the lines. LP females displayed a greater resource acquisition capacity than V females (regardless of the generation of selection), but only in highly demanding conditions (HC and NF). This pattern was already observed by Theilgaard et al. [24] for LP and V females. Conversely, 20 generations of selection to increase litter size at weaning was not accompanied by an overall increase in the female’s resource acquisition capacity. Selection programmes using crossbred rabbit females also showed that overall feed intake did not increase [42], although Quevedo et al. [43] and Savietto et al. [26] observed a different time-trend in the feed intake of crossbred and pure rabbit females from recent generations of selection, i.e. they had a higher intake during early lactation. This strategy combined with adequate management of body reserves is adapted to the hot summers of Mediterranean areas, where selection of line V is carried out. Moreover, Savietto et al. [26] reported that under HC and NF constrained conditions, V16 and V36 females presented a similar time-trend for DE intake and a similar resource acquisition capacity. Therefore, 20 generations of selection for litter size at weaning did not increase the environmental sensitivity of rabbit females with respect to resource acquisition capacity.

The higher resource acquisition capacity of LP females under challenging conditions seems to be the main strategy used by the founder females of this line to achieve a long and productive lifespan. In commercial farms, long productive lifespan is only attained by females that maintain their prolificacy (adequate litter size at birth and at weaning) over different reproductive cycles. In commercial farms, food is freely available throughout the females’ productive life. However, to meet their needs and adequately face any possible environmental constraints, females can develop different strategies. Accretion of body reserves is one possibility whereby females accumulate fat in non-limiting conditions to use it when the acquisition of resources is limited (e.g. hot seasons, poor food quality or even pathogens). This is a well-known strategy used by different farmed livestock species, to alleviate periods of nutritional stress (e.g. cows [44] or rabbits [18]). However, this strategy is probably not the most suitable to maximize fitness, since during their productive life, rabbit females may encounter expected but also unexpected constraints of different intensities, durations, and even frequencies. In addition, under conditions of unlimited food availability, females may gain fat and carrying excess body fat is costly, both in evolutionary [45] and metabolic terms [46]; indeed, some surveys have shown that excess body fat in rabbit females can be associated with an increased risk of being culled [47,48]. Bearing this in mind, the high resource acquisition capacity observed for LP females seems to be a more reasonable strategy to alleviate the negative effects of limiting conditions since it prevents excessive mobilization of body reserves, and thus favours a long productive lifespan. However, for this resource acquisition strategy to be effective, it needs to be associated with an adapted allocation of the resources among the competing life functions.

### Resource allocation strategies

Neither the different foundational criteria of LP and V females (i.e. LP *vs.* V36) nor the selection history of line V (i.e. V16 *vs.* V36) altered the females’ resource acquisition capacity in normal conditions. However, allocation of the resources differed. Figure 5A represents the percentage of energy acquired and allocated to different life functions during two consecutive reproductive cycles under the NC environment. We observed that, in productive females with a long life expectancy, resources are not wasted; instead a greater proportion of the obtained resources are assigned to produce milk, which is very important to ensure survival and development of the new-born kits [49], and for body reserves but at an adequate level to avoid the risks related with an excess of body fatness [45]. Moreover, only a very small amount of energy was allocated to stillborn kits, which is an interesting strategy to avoid risks related with late gestational loss [50].

After 20 generations of selection to increase litter size at weaning, V36 females were able to assign 0.155 MJ per parturition more than V16 females to produce viable kits (live born kits), thus reducing the expenses to non-viable kits (stillborn). García and Baselga [8] already reported an increased number of live born kits in response to selection for line V, and Quevedo et al. [43] observed a greater efficiency of DE use for foetal growth. Selection to increase litter size at weaning was also accompanied by higher milk yield in early lactation [26,43], which represents a critical period for the survival of kits [49]. These strategies had no negative impact on body fat reserves [43,47] through lactation [26]. However, there is evidence of a trade-off between the pregnant uterus and the mammary gland when rabbit females are concurrently pregnant and lactating [51]. In a selection context where fitness is attained by females that wean large litters in short intervals, selection of line V seems to favour females that can rapidly change the priority between the current (kits being nursed) and future litters (foetus development). In addition, in a selection design where the time-span during which females can show their genetic potential is limited (selection takes into account repeated measures on the same animal [27]) and that promotes different strategies to allocate the obtained resources, maintaining reproduction depended more on the accretion and use of body reserves [26].

An interesting aspect of our study is that the differences in adaptive capacities that the different lines have acquired and that contribute greatly to their robustness, were only revealed by comparing the lines under different environmental conditions. Understanding the strategy adopted by LP females to achieve a long productive lifespan was only possible by evaluating the females’ responses in constrained conditions. Similarly, the emergence of environmental sensitivity in response to selection, which is indicative of specialization, could only be observed under constrained conditions. The differences in life history strategies between LP and V36 females were highlighted under the HC environment. Previously, it was shown that LP females used body reserves to maintain their reproductive performance in the presence of constraints [20]. However, our results show that LP females also made use of their greater resource acquisition capacity to sustain reproductive performance. Intriguingly, this increased resource acquisition capacity was coupled with an adjustment in the allocation of these resources such that the investment in litter size was limited. Although LP females acquired more DE than V36 females, they did not invest the energy surplus in a larger litter size (see Figure 5B). They seem to be able to avoid the follow-on risks related with intense foetal growth by partitioning relatively less energy to produce non-viable kits than V36 females (e.g. LP females assigned only 8.5% of the 1.6 MJ used for litter production to produce stillborn kits while V36 females assigned 17.3% of the 1.5 MJ used for litter production to produce stillborn kits). Instead, the greater DE intake capacity was partitioned into milk yield, which ensured the survival of kits, without reserving additional resources to maintain the fat level to values similar to those observed under the NC environment. For LP females, it seems that fat reserves are a safety factor, rather than a necessary energy surplus to ensure reproduction [25].

The advantage obtained by V36 females in response to selection for litter size after 20 generations was observed under constrained conditions, especially under the HC environment. V36 females reduced milk yield and safeguarded body condition (i.e. live weight and PFT), which led to a small decrease in total number of kits born (−1.9 kits per parturition compared to the NC environment). However, the number of live born kits was smaller under constrained conditions compared to normal conditions (−3.4 kits per parturition). In contrast, under constrained environments, V16 females maintained milk yield to levels similar to those under the NC environment, which increased the relative partition to the current litter. V16 females also displayed a decreased gestational effort (i.e. −2.6 kits born in total per parturition, compared to the NC environment) which means that the available resources were adjusted to give birth to viable kits. Contrary to what was observed under the HC environment, V36 females under the NF environment partitioned the energy resource that was not aimed at producing milk into gestation to ensure a small reduction in litter size (−1.4 total kits born per parturition compared to the NC environment) but more viable kits (+1.3 live born kits per parturition).

Our work provides evidence of changes in the resource acquisition capacity and the allocation of obtained resources into different life functions as a result of different selection criteria in two maternal rabbit lines. Line LP was founded by selecting females with a long productive lifespan from commercial farms [21] where culling and early mortality are the main factors that limit lifespan. A long productive life is, thus, an indirect indicator of successful adaptation to variations in environmental conditions (for other examples see [52,53]. Reproduction has a cost [54] and there is a consensus on the existence of a trade-off between reproduction and lifespan [55]. However, trade-offs also depend on the environmental conditions [23,55], thus the selection of species or lines that can obtain and partition resources into competing functions depends on the interaction between genotype and environment [23]. Our work demonstrates that this is the case for LP females. They did not prioritize a single function, but had an increased acquisition capacity, allocating resources to sustain prolificacy without increasing excessive mobilization of body reserves. In this case, fat reserves are a safety factor.

**Figure 6:**
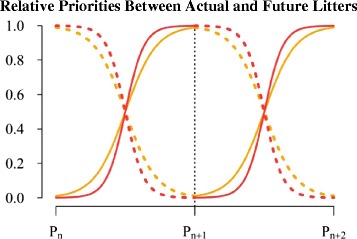
**Relative priorities between the current and future litters in rabbit females.** Scheme representing the changes in the relative priorities between the litter being nursed (dotted lines) and the litter under gestation (solid lines) for V16 (orange) and V36 (red) females; two consecutive cycles are represented. Twenty generations of selection for litter size at weaning resulted in females that are able to change priorities rapidly between the current and future litters (reprinted with permission of Pascual et al. [18]).

Selection to increase litter size at weaning over 20 generations did not favour a greater acquisition capacity of female rabbits, but the selection criteria were attained by female rabbits that proportionally assigned more resources to favour litter size at weaning. The main reason for not favouring a greater resource acquisition capacity seems to be related to the change in priorities between the current and future litters, as suggested by [45]. Several arguments support this hypothesis i.e.: the limited time span during which females can express their genetic potential together with the evidence available from the literature on the competition between the gravid uterus and the mammary gland [51], the larger litter size at birth with a relatively high milk yield during early lactation and low milk yield during late lactation [26,43] and the greater dependence of V36 females on fat reserves to ensure reproduction [26]. A schematic representation of the hypothesis that V36 females allocated resources to functions related to the rapid change in priority between litters, rather than to increasing resource acquisition, is in Figure 6 (reprinted from Pascual et al. [18]). Additional research on how time limitations affect the temporal pattern of the allocation of resources (under non-limiting conditions) between life functions is necessary.

### Ethical statement

The Research Ethical Committee of the Universitat de Politècnica de València gave ethical approval for the protocols of this research.
